# Exploring usage and perceived effectiveness of fitness trackers and mobile health applications among university students in Saudi Arabia

**DOI:** 10.1038/s41598-025-22743-3

**Published:** 2025-10-27

**Authors:** Naji Alqahtani, Adel Bashatah, Mohammad K. Alharbi, Wajid Syed

**Affiliations:** 1 Department of Nursing Administration and Education, College of Nursing, King Saud University, Riyadh, 11451 Saudi Arabia; 2 Department of Clinical Pharmacy, College of Pharmacy, King Saud University, Riyadh, 11451 Saudi Arabia

**Keywords:** Fitness trackers, Mobile health, Physical activity, Health and wellness, Nursing students, Healthcare, Health care, Medical research, Risk factors

## Abstract

The integration of technology into health and wellness has been gaining momentum. Wearable technology and mobile health applications (Apps) have become increasingly popular for tracking health-related behaviors. This study explores the usage, perceived impact, and tracking capabilities of fitness trackers (FTs) and mobile health Apps among university students in Riyadh, Saudi Arabia, over a 4-month period in 2024. A cross-sectional, web-based study was conducted in the Riyadh, region of Saudi Arabia, between March and June 2024. The target population was undergraduate healthcare students enrolled in the faculties of nursing, pharmacy, and EMS at a public university in Riyadh for the academic year 2024. The questionnaires collected information on demographics and FT usage, perceptions. The other sections focused on Mobile Health App usage, reasons for not using, the most commonly utilized feature, source of FTs. The last section assessed the impact of mobile apps and FTs on health. A total of 523 healthcare students responded to the study by giving a response rate of 95.1%. However, the analysis included on 357 undergraduate students who used FTs and mobile health apps. The prevalence of use of FTs and mobile health apps was 68.3%, among those 40.1%(*n* = 143) of them were belongs to pharmacy, 30.3% (*n* = 108) of them were EMS and 29.7% (*n* = 106) of them were nursing. Among the users, the most popular FT was wristband 43.7% (*n* = 156) smart watches 35.9% (*n* = 128). In addition, 52.1% (*n* = 186) of the healthcare students reported using mobile health Apps. Healthcare students used FTs to increase physical activity 24.4%, improving workouts 21.6%, monitoring heart rate, and losing weight. In addition, 48.1% of the healthcare students agreed that FTs increases their physical activity. This study revealed fourth-year students were more likely to wear FTs every day compared to their peers, (*p* = 0.022; χ² =29.223), and athletes used FTs significantly more frequently than non-athletes (*p* = 0.022; χ² = 11.447) suggesting significant association between year of study and student’s athlete’s status and FTs use. Our findings emphasize the importance of technology in promoting health and wellness, especially through the use of FTs and mobile health apps. Significantly, the use of this technology was linked to higher levels of physical activity and increased confidence, illustrating the necessity for raising awareness among students and individuals regarding the advantages of FTs and mobile health apps. Additional research is needed to investigate the reasons behind utilizing technology for health enhancement, ultimately guiding efforts to maximize its benefits.

## Introduction

The integration of technology in health and wellness is rapidly evolving^1,2^. Fitness trackers(FTs) and mobile health apps have gained significant attention for tracking physical activity, calorie intake, weight management, and other health behaviors^1,2^. A strong link exists between healthy behaviors, such as regular exercise and balanced eating, and positive health outcomes^2,3^. The World Health Organization (WHO) recommends at least 150–300 min of moderate exercise per week^4^.To help achieve this, technological innovations, FTs are becoming increasingly popular worldwide^5^. These devices track progress and motivate users to improve their performance while maintaining a higher peak heart rate for a brief period^6^. Studies have shown that, on average, individuals who use physical activity trackers increase their daily step count and their weekly physical activity^5,6^.

The global wearable technology market is set for substantial growth, projected to reach 1.1 billion users by 2025, driven predominantly by the fitness and wellness sector^7,8^. Moreover, the worldwide smart wearable device market is expected to reach USD 528.7 billion by 2036, expanding at a compound annual growth rate (CAGR) of 19.7% from 2024 to 2036 ^9^. As of 2023, the global smart wearables market was valued at USD 64.9 billion, with wrist wear accounting for a dominant 35% market share^8–10^. The government’s Vision 2030 transformation program has fueled rapid growth in the digital health sector in Saudi Arabia. The Kingdom has made significant investments in digital health initiatives with the goal of improving healthcare services and increasing accessibility^1,11,12^. Among the major projects are the introduction of several mobile applications, such as the Sehhaty App^13^. This app has over 30 million users and provides a platform for managing appointments, medications, and personal health information^13^. The Seha Virtual Hospital (SVH) is a telemedicine network that offers specialized healthcare services to more than 123,000 beneficiaries across 170 facilities^14^. Additionally, Wasfaty is a platform that links pharmacists with primary care clinics and hospitals^15–17^.

In Saudi Arabian wearable technology market generated significant revenue in 2023, reaching USD 626.0 million, with projections indicating a substantial increase to USD 2,485.8 million by 2030^18,19^. This growth is expected to be driven by a CAGR of 21.8% from 2024 to 2030. Additionally, FTs emerged as the top revenue-generating goods in 2023, reflecting their widespread adoption both locally and globally^18,19^. The adoption of wearable FTs and mobile health applications varies significantly across countries and populations. Prevalence rates differ substantially, with studies reporting 26.5% among Lebanese university students^3^ and 22.5% among college students^20^. Globally, FT use ranges from 22.3% in Japan to 38.2% in the United States and Europe, where 59.9% of individuals utilize mobile health apps^21^. In Saudi Arabia, a regional study found that 57.5% of adolescents own a FT, highlighting diverse adoption patterns^22^. The most commonly cited reasons for using FTs and mobile apps include promoting physical activity, fostering healthy habits, and enhancing overall well-being. These digital tools offer an accessible way to track progress, set goals, and maintain motivation, ultimately creating a sense of community and encouraging friendly competition^1,3,8,23–25^. The adoption and utilization of FTs can be explained by theoretical frameworks such as the Technology Acceptance Model (TAM)^26^ and the Health Belief Model (HBM)^27,28^. According to TAM, students are more likely to accept and use FTs if they perceive them as useful for tracking fitness, setting goals, and improving health^26,28^. Moreover, the tracker’s ease of use and user-friendly interface significantly influence its adoption^26,28^. Conversely, complexity or difficulty in using the tracker may deter students from utilizing tithe HBM suggests that students who perceive themselves as vulnerable to health issues, such as obesity or physical inactivity, and believe that FTs can provide health benefits, are more likely to adopt and use these devices^27,28^. **Figure-**1 shows theoretical framework based on the TAM.

**Fig. 1:**
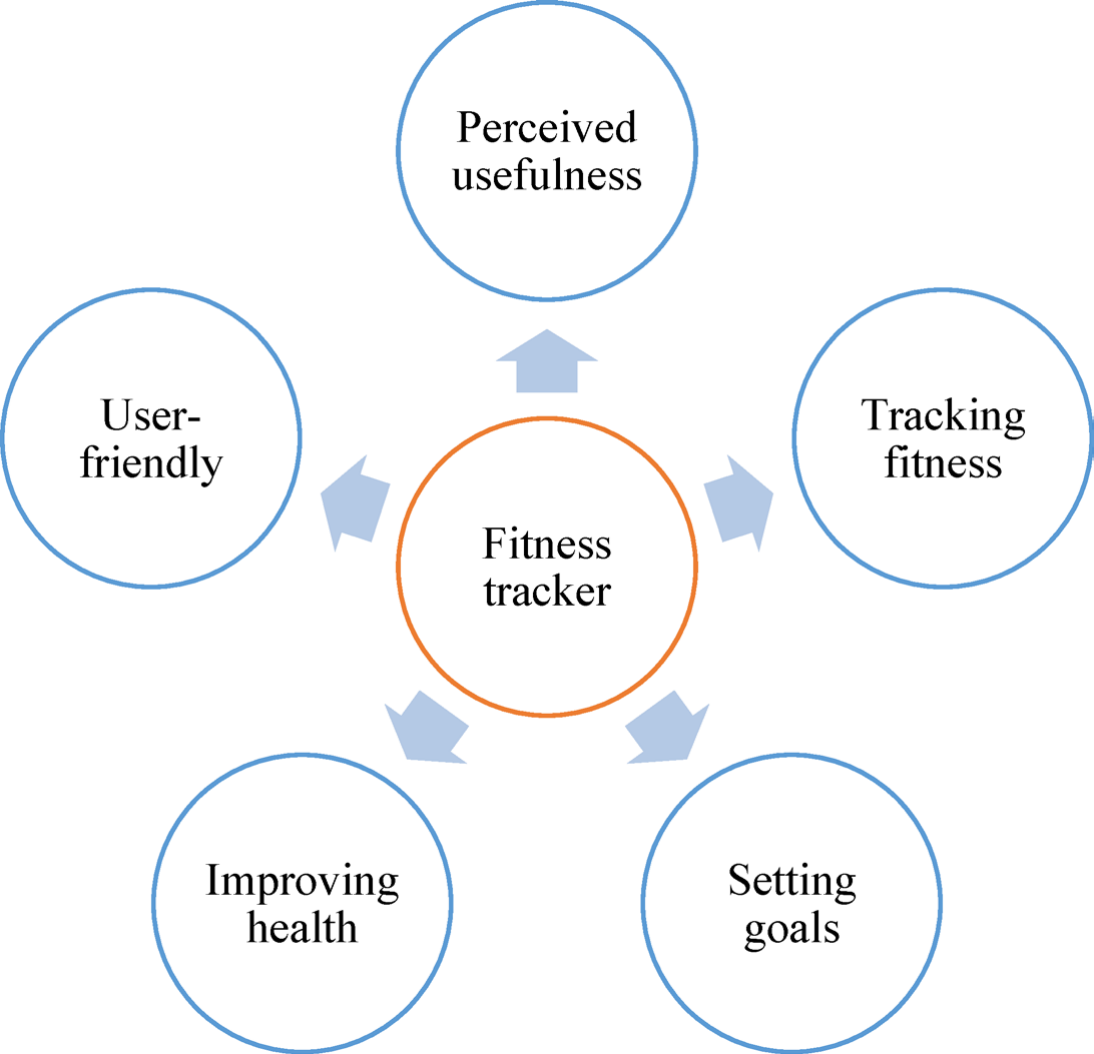
Theoretical framework based on the Technology Acceptance Model (TAM).

### Commonly used mobile health apps

Despite their benefits, FTs and mobile health apps also contribute to reducing the need for expensive medical interventions by monitoring heart rate, sleep quality, and promoting physical exercise and self-management of chronic illnesses^24,29,30^. Moreover, FTs can motivate inactive individuals to start exercising^5^ The growing prevalence of chronic diseases, such as obesity, diabetes, and heart disease, drives the demand for FTs and mobile health apps, as consumers seek to proactively manage their health^31^. Advanced features like GPS tracking, heart rate monitoring, and sleep tracking have increased the appeal of FTs, while digital health platforms and mobile apps have facilitated data analysis and interpretation, enabling informed decision-making about health and fitness goals. However, research on the utilization of FTs and mobile health apps in Saudi Arabia is limited, particularly regarding students and individuals’ perceptions. In addition, only 54.6% of university students engage in regular physical activity, with high sedentary behavior^32–34^. Interestingly, 63% of younger adults in Saudi Arabia are physically active compared to others^35^. However, healthcare students face unique challenges, including heightened academic stress and time constraints, which can hinder their ability to maintain a healthy lifestyle^36,37^. The habits formed during formative years tend to persist into later life. If students fail to adopt healthy habits during their college years, the likelihood of adopting them later in life decreases significantly^36–38^. The consequences of unhealthy habits and obesity in young adults are far-reaching, including - type II diabetes, hypertension, and cardiovascular disease risk factors^36–38^. As future healthcare professionals, healthcare students offer a unique perspective on the benefits and limitations of FTs and mobile health applications^36,37,39^. Additionally, being in a transitional phase, balancing academics with social and personal life, may influence their health behaviors and technology adoption^36,37,39^. Examining their views can provide valuable insights into future trends in healthcare practice and patient care. This study aimed to investigate the usage, perceived impact, and tracking capabilities of FTs and mobile health applications among university students in the College of EMS, Nursing, and Pharmacy in Riyadh, Saudi Arabia, over a 4-month period in 2024.

## Methods

**Fig. 2:**
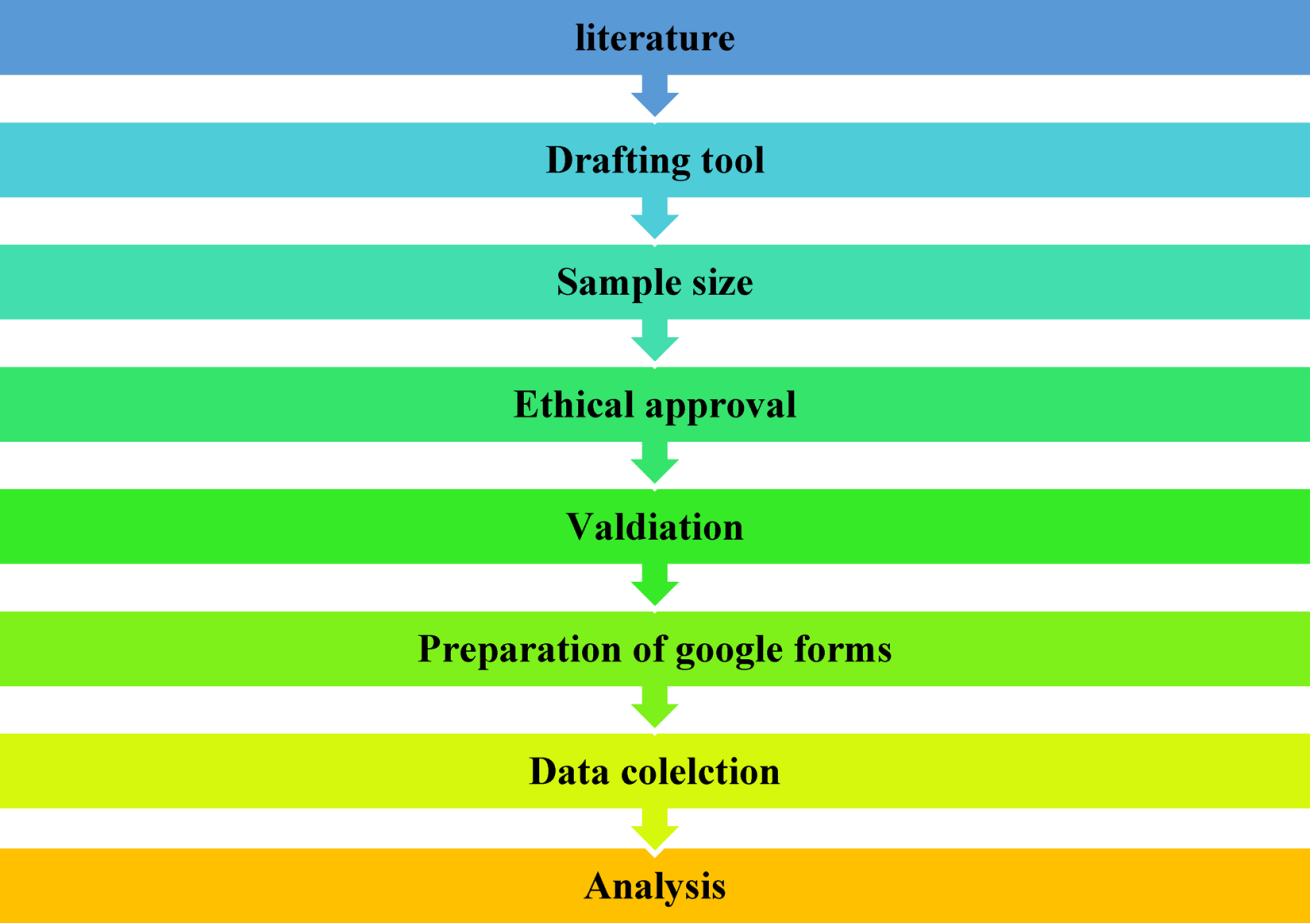
Flow diagram of the study.

A cross-sectional, web-based study was conducted in the central region of Saudi Arabia, Riyadh, between March and June 2024. A stratified sampling approach was chosen and targeted undergraduate from specific colleges (Emergency Medicine (EMS), Nursing, and Pharmacy) using a survey at a public university in Riyadh during the academic year 2024. The study included undergraduate healthcare students enrolled in the Pharmacy, Nursing, and EMS faculties, Saudi students, living in the Riyadh region, regular campus attendees, and students, able to provide informed consent. Graduated students, students who completed their courses, or left their courses without finishing previous years, and students who did not use FTs and Mobile applications were excluded from the study. Furthermore this study was fallowed the Checklist for Reporting Results of Internet E-Surveys (CHERRIES)^40^.The detailed study procedure steps were presented in Fig. 2.

### Sample size

Similar to earlier studies^37,41,42^ the sample size for this study was calculated using Raosoft sample size calculator based on a 5% margin of error, a 95% confidence interval (CI), population of 20,000 students at study site and a 50% expected response rate^43^. The estimated sample size was 377 students, adjusting for the projected 10% attrition, the estimated final sample size is at least 414. Although to improve the study’s statistical power and allow for more accurate estimates and detection of significant differences and to minimize sampling bias and error, seeking cooperation from students in the study site to take part in the research, we were able to collect a total of 550 responses.

#### Ethics approval and consent to participate

Prior to data collection, ethical approval was obtained from the King Saud University Institutional Review Board at the Human research ethics committee (KSU-HE-24–905) in Riyadh, Saudi Arabia. All methods were conducted in accordance with the relevant guidelines and regulations. Additionally, informed consent was obtained from the students. They were informed and assured that their responses would be kept confidential. No individuals were pressured to provide an answer, and students had the right to withdraw from the research at any time.

### Data collection tool

The questionnaire was adapted from existing literature^3,20^and consisted of Six sections. Section one captured student demographics and health information, including sex, age, professional classification, year of study, student-athlete status, chronic diseases and there was a question asking about the use of FT along with mobile health apps. The section two explored Summary of FTs usage, including, length of use, most commonly used FTs, frequency and occasions of use, reasons for use and information collected, confidence in physical activity since using a FT, perceived impact on physical activity levels, daily step count based on tracker data. The section three about utilized features of FTs and mobile health apps. The section four about commonly used Mobile Health Apps and its frequency. On the other section five collected information on the reasons and causes for discontinue using FTs and Mobile Health Apps. The last section collected information on the source for the FTs and Mobile Health Apps use and Perceived impact on the health. The impact on health was consisted four questions assessed on the 6-point rating scale.

The initial questionnaire draft underwent content validation by a panel of experts, comprising three professors from the College of Nursing and a researcher, who independently assessed its appropriateness and validity. A pilot study was then conducted with a random sample of 30 undergraduate students to ensure linguistic and conceptual clarity. The pilot study results were excluded from the main investigation. The questionnaire’s reliability was evaluated using Cronbach’s alpha, yielding an overall coefficient of 0.71, which indicated acceptable reliability. The questionnaire was then converted into an electronic version using Google Forms and distributed via social media platforms like WhatsApp and email. Informed consent was obtained from all students before completing the survey. Data was collected using online questionnaires distributed to students in the College of EMS, Nursing, and Pharmacy. A stratified sampling approach was employed, targeting specific student populations within these colleges, the online survey methodology allowed for a representative sample of students from each college to participate.

### Statistical analysis

The acquired data was analyzed using IBM SPSS Statistics 26 software from IBM Inc. in Chicago, IL, USA. The data were analyzed using descriptive statistics to summarize the demographic characteristics of the participants and their use of FTs and mobile health apps. Frequency distributions (n) and percentages (%) were calculated for categorical variables, while means with standard deviations were calculated for continuous variables. The results of Shapiro–Wilk and Kolmogorov–Smirnov test, shows a p value of 0.751, suggesting that data fallowed normal distribution. To examine the association between FT and Mobile Health App usage frequency and student characteristics, Chi-Square or Fisher’s exact test was employed at a significance level of *p* < 0.05.

## Results

### Demographic characters

Out of 550 healthcare students surveyed, 523 responded to questionnaires (95.1% response rate). However only 357 undergraduate students were currently using FTs along with mobile health apps, representing a 68.3% usage rate among respondents and therefore only 357 students were included in the final analysis. Significant differences were found between FT users and non-users regarding sex (*p* = 0.047). Demographic characteristics are detailed in Table 1. The sample comprised students from various health disciplines including students from EMS (30.3%, *n* = 108), nursing (29.7%, *n* = 106), and pharmacy (40.1%, *n* = 143). Most students were in their fourth year (23.5%, *n* = 84), with a mean age of 24.92 ± 2.68 years. The sex distribution was nearly equal, with 50.1% males (*n* = 179) and 49.9% females (*n* = 178).

**Table 1:** Demographic health characters of the students according to professional classification (*n* = 523).

Estimation of prevalence				
Total sample		523	Prevalence Total/Users 523/357= 68.3%	
Characteristics		Users *n*(%) 357 (68.3%)	Non users *n*(%) 166(31.7%)	*p* value
**Sex**	Male	222(71.6)	88(28.4)	0.047
	Female	135(63.4)	78(36.6)	
**Professional classification**	Pharmacy	141(74.2)	49(25.8)	
	Nursing	143(65.6)	75(34.4)	0.081
	EMS	73(63.5)	42(36.5)	
**Year of study**	1 st year	68(66.7)	34(33.3)	
	2nd year	58(71.6)	23(28.4)	
	3rd year	68(62.4)	41(37.6)	0.372
	4th year	84(67.2)	41(32.8)	
	Interns	79(74.5)	27(25.5)	
**Chronic diseases**	Yes	74(72.5)	283(67.2)	0.300
	No	28(27.5)	138(32.8)	
**Are you a student athlete?**	Yes		83(50)	
	No	165(46.2) 192(53.8)	83(50)	0.420

### Summary of fitness tracker usage

Regarding duration of use, 20.7% (*n* = 74) of students had used their FTs for 4–6 months. The most popular FT type was wristbands (43.7%, *n* = 156), followed by smartwatches (35.9%, *n* = 128). In terms of wear frequency, 23% (*n* = 82) of users wore their trackers daily, while 21.6% (*n* = 77) wore them most days. The primary contexts for wearing FTs were during workouts (43.3%, *n* = 155) and daytime activities (30.8%, *n* = 108). The main purposes for using FTs and mobile health apps were to increase physical activity (24.4%), monitor heart rate (16.2%, *n* = 58), track health conditions (11.2%, *n* = 40), and support weight loss (13.7%, *n* = 49). Regarding the impact of FTs on physical activity, 24.6% (*n* = 88) of students reported a significant increase. Based on tracker data, 20.2% (*n* = 72) of students regularly achieved 10,000–12,499 steps per day. Detailed usage patterns are presented in Table 2.

**Table 2:** Summary of fitness tracker usage among students (*n* = 357).

Variables	Frequency (*n*)	Percentage (%)
How long have you been using the tracker?		
< 1 month	67	18.8%
1–3 month	49	13.7%
4–6 month	74	20.7%
7–9 month	50	14.0%
10–12 month	55	15.4%
> 1	62	17.4%
Which wearable fitness tracker do you currently use? Wristband	156	43.7%
Smart watch	128	35.9%
Other	73	20.4%
How often do you wear your fitness tracker?		
A few times a month	62	17.4
A few days a week	63	17.6
Everyday Most Days	82 77	23.0% 21.6%
When I remember	73	20.4%
When do you wear your fitness tracker?		
All day and while sleeping	94	26.3%
Only during the day	108	30.3%
Only when working out	155	43.4%
Use of fitness trackers and mobile health apps		
To improve workouts	77	21.6%
To Increase physical activity	87	24.4%
To lose weight	49	13.7%
To monitor my sleep	32	9.0%
To monitor my heart rate	77	21.6%
Other	35	9.8%
Which information is the most important to you?	48	13.4%
Number of stairs Climbed	73	20.4%
Number of Steps	118	33.1%
Active Minutes	48	13.4%
Calories burned	70	19.6%
Distance/Miles		
How would you rate your confidence to be physically active since you began using a fitness tracker?		
Not confident	84	23.5
Extremely Confident	92	25.8%
Moderately Confident	102	28.6%
Slightly Confident	79	22.1%
Does using a fitness tracker increase your physical activity?		
Not at all	0	0
A little bit	63	17.6%
Not sure	60	16.8%
Quite a bit	62	17.4%
Somewhat	84	23.5%
Very much	88	24.6%
Do you feel more motivated to be physically active because of your fitness tracker?		
Not at all	50	14.0%
A little bit	73	20.4%
Not sure	59	16.5%
Quite a bit	58	16.2%
Somewhat	64	17.9%
Very much	53	14.8%
Does using a fitness tracker increase the number of steps you take every day?		
Not at all	53	14.8%
A little bit	57	16.0%
Not sure	42	11.8%
Quite a bit	60	16.8%
Somewhat	71	19.9%
Very much	74	20.7%
Based on your tracker data, about how many steps do you get in on regular basis.		
Less than 5,000 Steps per day		
5,000–7,499 Steps per day		
7,500-9,999 Steps per day	54	15.1%
10,000–12,499 Steps per day	61	17.1%
12,500 or more Steps per day I don’t Know	53 72	14.8% 20.2%
	60 57	16.8% 16.0%

### Utilized features of fitness tracker’s and mobile health apps among users

The most valued feature of FTs and mobile health apps was step tracking (39.2%, *n* = 140). **Figure-**3 illustrates the frequency of healthcare students’ responses regarding utilized features of FTs and Mobile health apps.

**Fig. 3:**
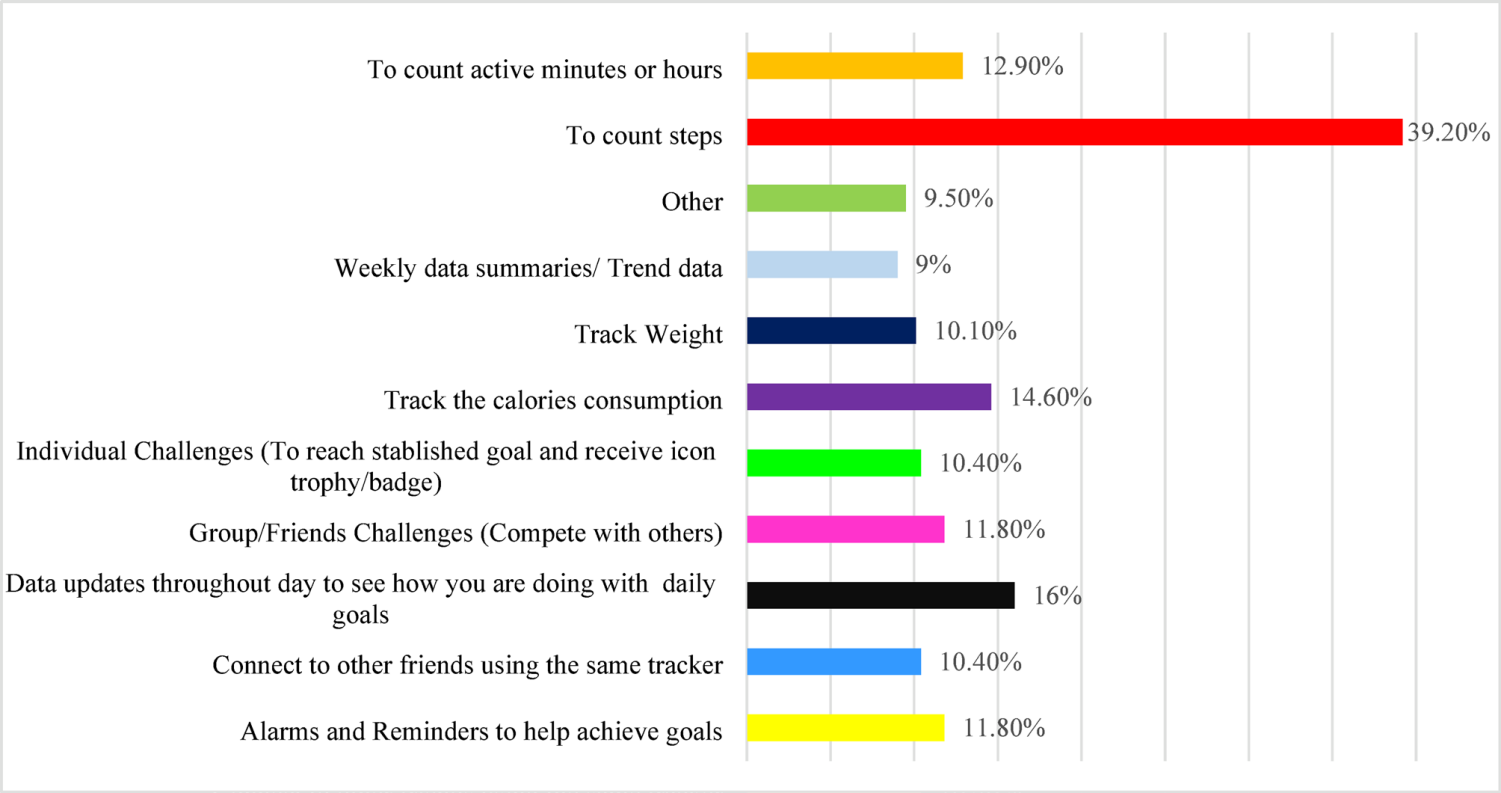
Utilized Features of Fitness Tracker’s and mobile health apps among users (*n* = 357), (Multiple answers were allowed).

### Commonly used mobile health apps

The most popular Apps were Lose It (15.1%, *n* = 54) and My Gym/Health and Weight Watchers (13.7%, *n* = 49). Regarding usage frequency, 15.1% (*n* = 54) of students used mobile health apps with their FTs once daily, 13.7% (*n* = 49) used it five times daily, and 12.9% (*n* = 46) used it three times daily (Table 3).

**Table 3:** Mobile health app usage (*n* = 357).

Variables	Frequency (*n*)	Percentage (%)
Commonly used mobile health apps		
Lose it	54	15.1%
My fitness Pal	44	12.3%
My gym/health Social media (YouTube/Instagram/Facebook)	49 210	13.7% 58.9%
How often do you use mobile health app?		
Once a week	41	11.5%
A five times in a week	47	13.2%
Three times in a week	46	12.9%
Two times once a week	46	12.9%
A five times a day	49	13.7%
Three times in a day	46	12.9%
Two times in a day	30	8.4%
Once in a day	54	15.1%

### Reasons for discontinuing fitness trackers and mobile health apps

**Fig. 4:**
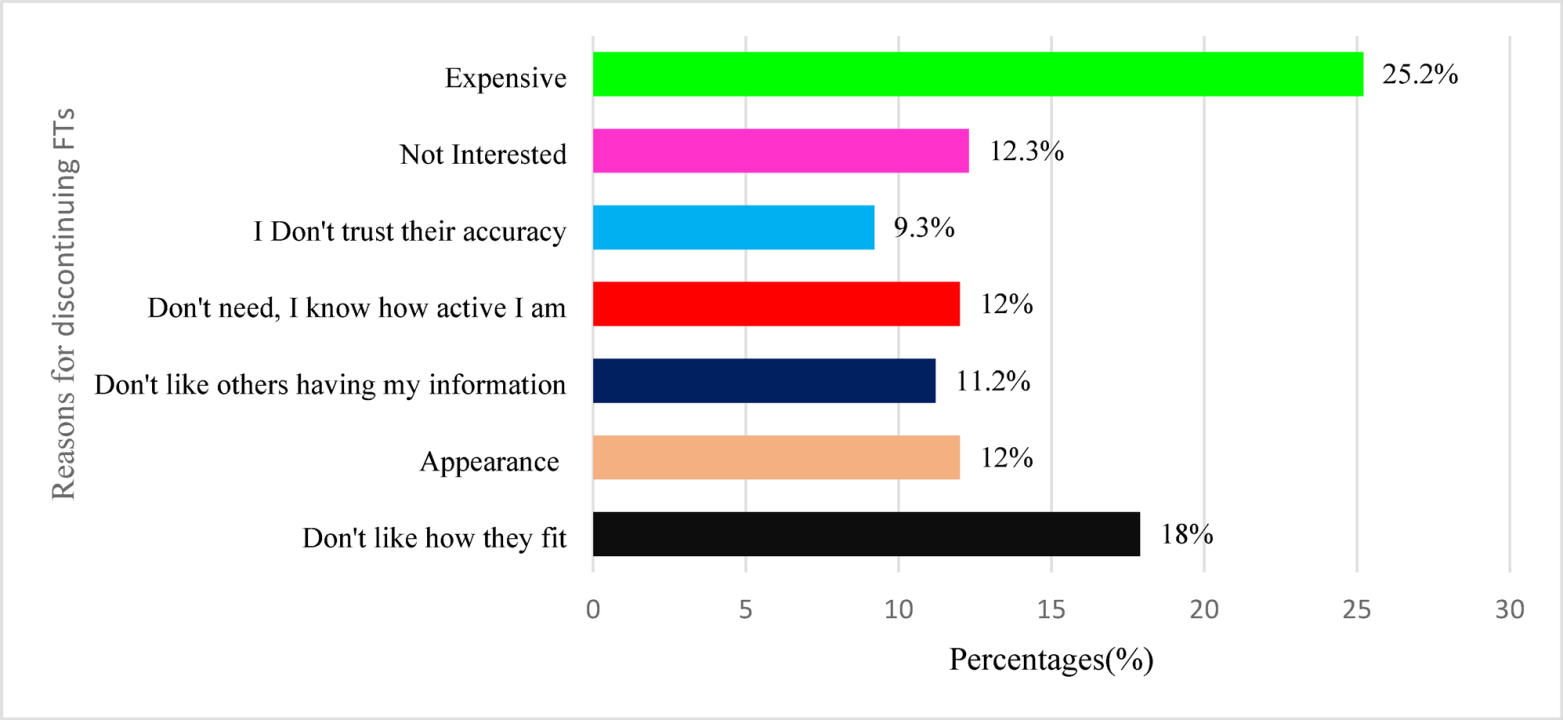
Reasons for Discontinuing Fitness Trackers among Users (*n* = 357).

Students cited various reasons for not wearing FTs, including discomfort (18%, *n* = 64) and aesthetic concerns (12.0%, *n* = 43). Privacy concerns were also a factor, with 11.2% (*n* = 40) of students hesitant to share personal data and 12.0% (*n* = 43) feeling confident in their activity level without tracking. Additional reasons included lack of interest (12.3% *n* = 44) and cost concerns (25.2%, *n* = 90), as illustrated in Fig. 4.

**Fig. 5:**
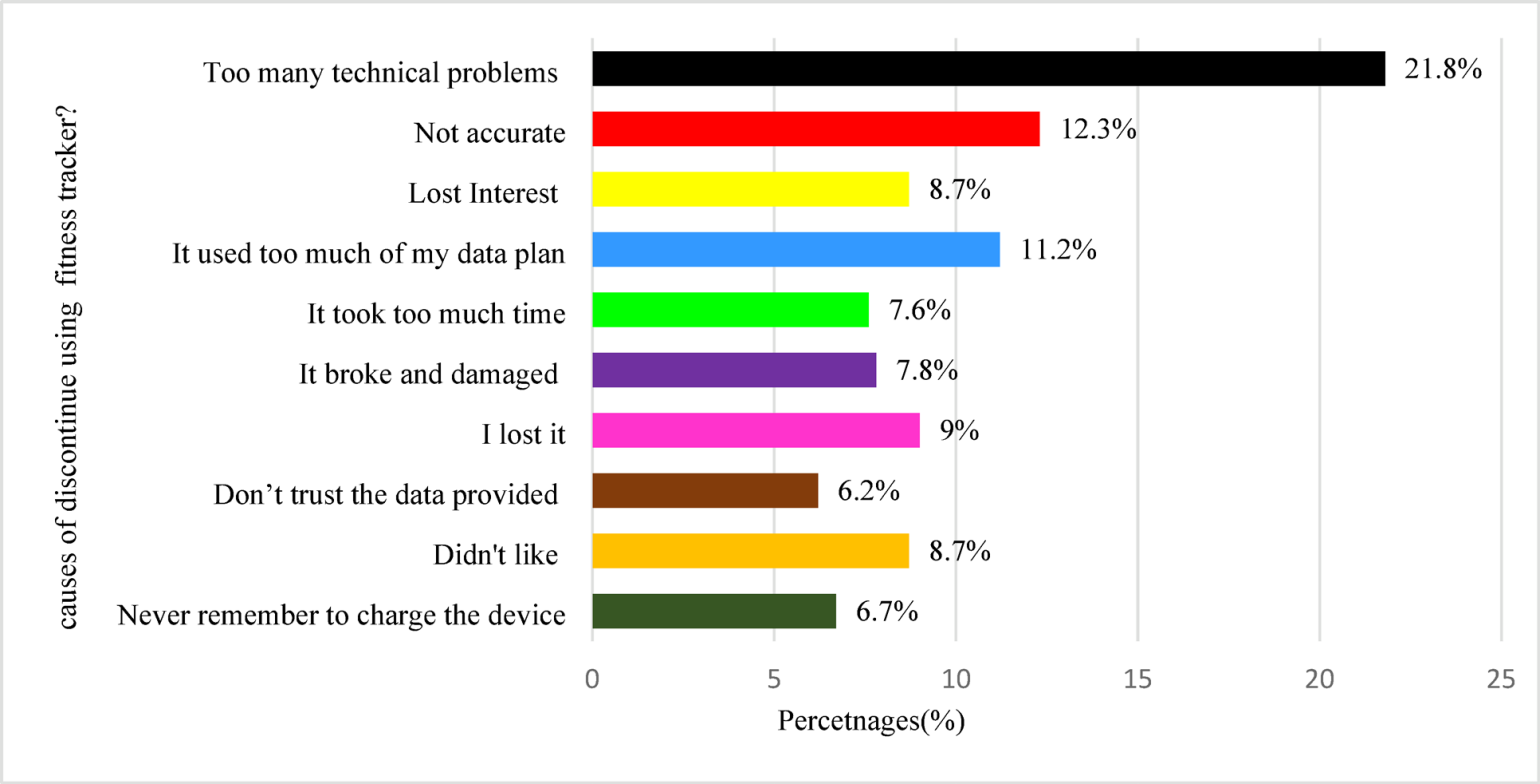
Reasons for discontinuing mobile health apps among users.

The primary reasons for discontinuing Mobile health apps were too many technical problems (21.8%), inaccurate data (12.3%, *n* = 44), consuming too much data 11.2% as shown in **Figure**-5.

### Source of information for the FTs and mobile health apps

**Fig. 6:**
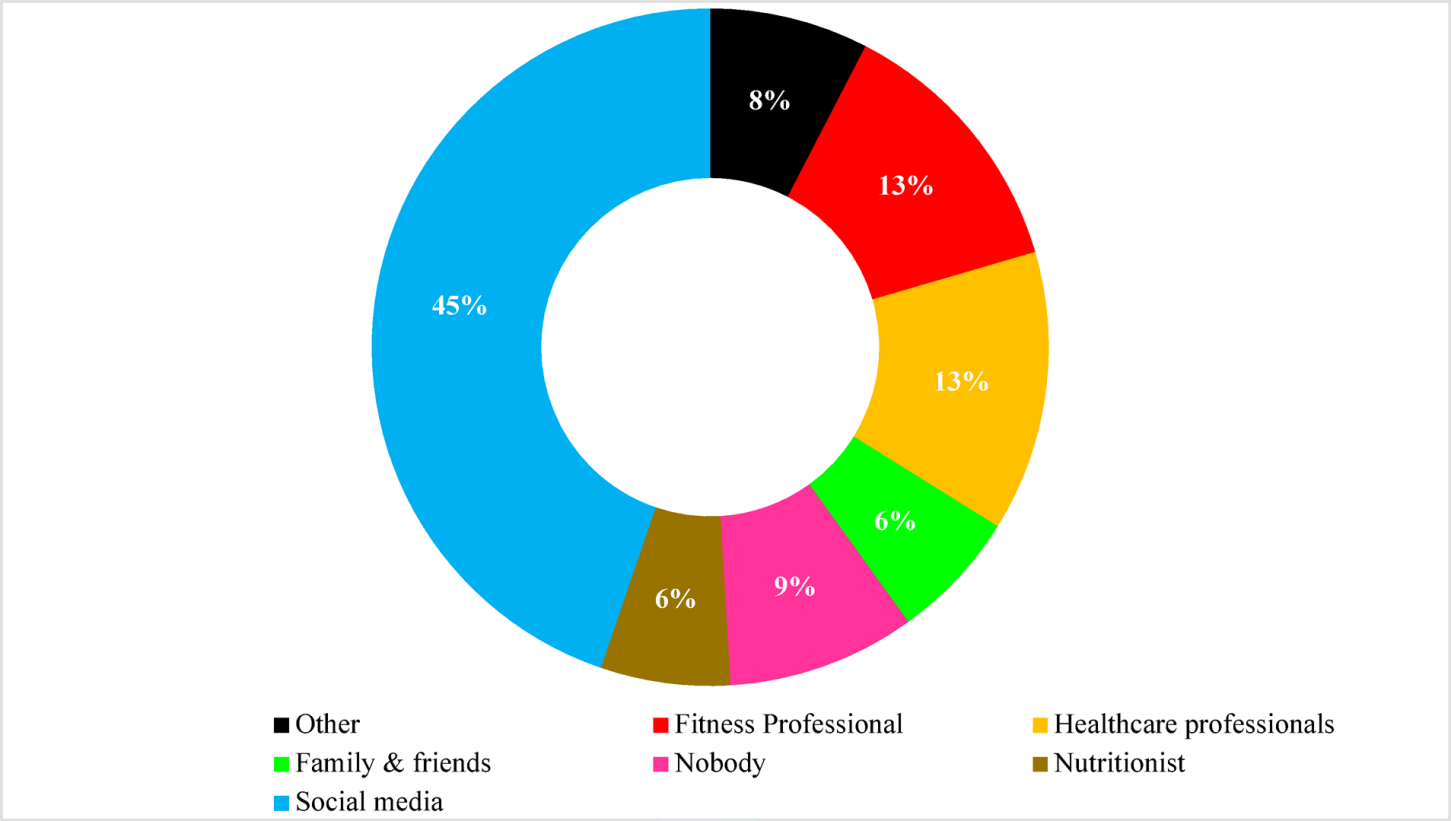
Source of information for the FTs and mobile health apps adoption among students

The primary sources of FT and mobile health apps adoption among healthcare students were social media (45%), followed by fitness professionals (13%), and healthcare professionals. Additionally, 6% of students reported family and friends as their source of influence (Fig. 6).

### Perceived impact of mobile health apps and fitness trackers on the health

**Fig. 7:**
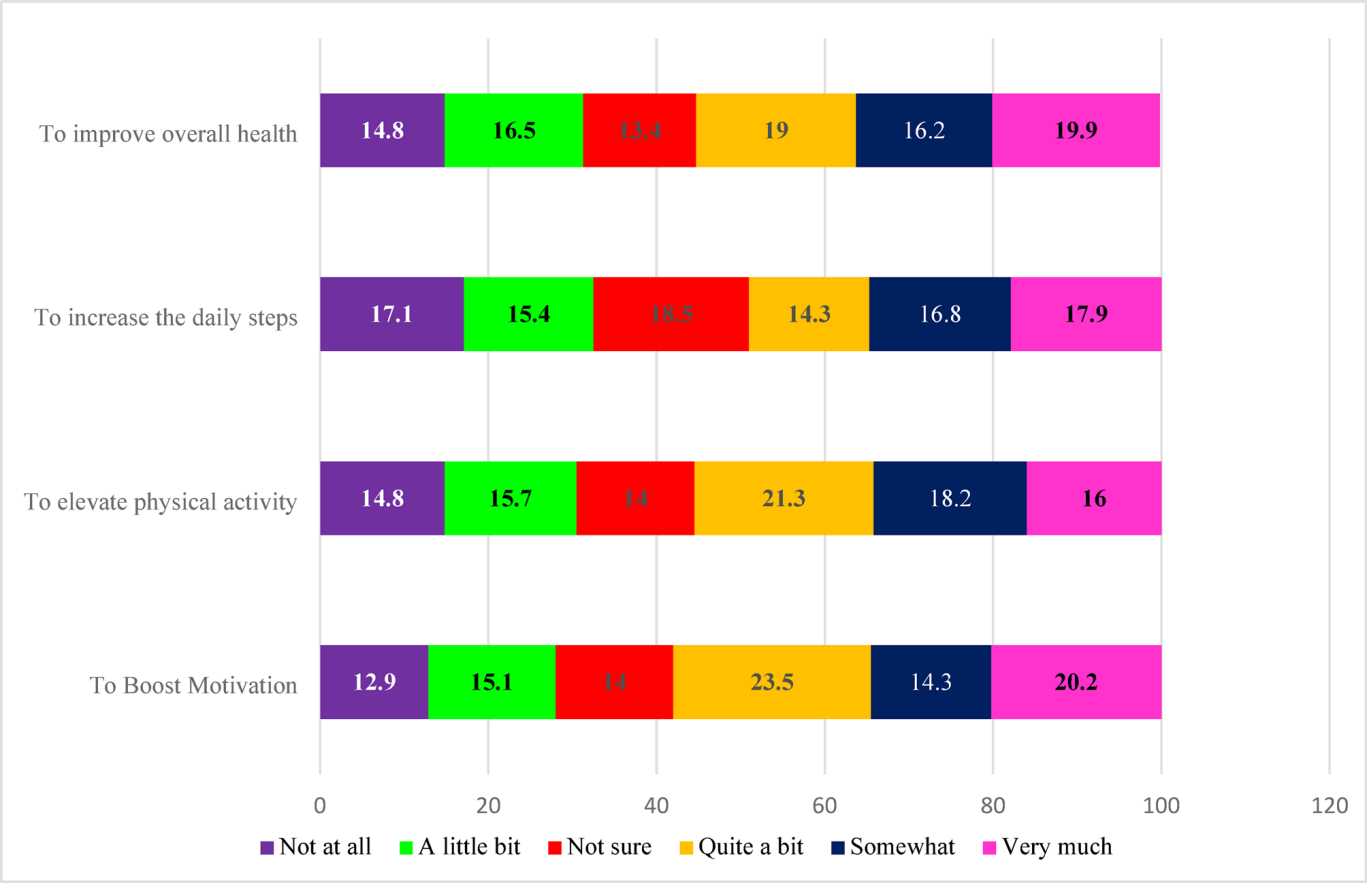
Perceived impact of mobile apps and fitness trackers on the health.

This study found that 58% of students believed FTs and mobile health apps boosted their motivation, while 55.1% reported improved overall health. Additionally, 55% of students indicated that FTs increased their physical activity, as illustrated in Fig. 7.

### Association between frequency of wearing FTs and student’s characters

This study revealed a significant association between the year of study and the frequency of wearing FTs. Specifically, fourth-year students were more likely to wear FTs every day compared to their peers, while interns wore FTs most of the time, surpassing first-, second-, and third-year students (*p* = 0.022; χ² =29.223). These findings suggest that the year of study is a significant factor influencing the adoption of wearable technology (Table 4). In contrast, the age of the student (*p* = 0.466; χ² = 3.577) and student classification (*p* = 0.925; χ² = 3.140) were not significantly associated with the use of wearable technology (Table 4). Furthermore, no significant association was found between frequency of FT use and sex (*p* = 0.470; χ² =), student classification (*p* = 0.927; χ² =3.140) presence of chronic diseases (*p* = 0.603; χ² =2.765). However, athletes used FTs significantly more frequently than non-athletes (*p* = 0.022; χ² = 11.447). In addition, the current analysis revealed no significant associations between mobile health app usage frequency and sex (*p* = 0.808, χ2 = 3.782), professional classification (*p* = 0.131, χ2 = 19.964), year of study (*p* = 0.158, χ2 = 35.426), athletic status (*p* = 0.173, χ2 = 10.288), or presence of chronic diseases (*p* = 0.763, χ2 = 4.195).

**Table 4:** Association between frequency of wearing FTs and student’s characters.

How often do you wear your fitness tracker?		Year of study?					Total	*p* value
		1st	2nd	3rd	4th	Interns		
A few days a week	Count	11	14	15	10	13	63	χ2 = 29.2230.022
	%	17.5%	22.2%	23.8%	15.9%	20.6%	100.0%	
A few times a month	Count	14	3	18	14	13	62	
	%	22.6%	4.8%	29.0%	22.6%	21.0%	100.0%	
Everyday	Count	15	10	11	31	15	82	
	%	18.3%	12.2%	13.4%	37.8%	18.3%	100.0%	
Most Days	Count	15	14	11	14	23	77	
	%	19.5%	18.2%	14.3%	18.2%	29.9%	100.0%	
When I remember	Count	13	17	13	15	15	73	
	%	17.8%	23.3%	17.8%	20.5%	20.5%	100.0%	

* Pearson Chi-Square.

## Discussion

### Prevalence of FTs and mobile health apps

The current analysis of healthcare students revealed that 68.3% used FTs and mobile health apps. These findings are consistent with previous studies^3,21,44,45^. For instance, Bardus et al. reported that 40% of students in Lebanon, used mobile health apps, while 26.5% used FTs^3^. Similarly, another study conducted at the University of Debrecen found 26.3% of students used FTs^46^. While a United States, a study with a sample of 356 college students reported 22.5% of college students used FTs^20^. In contrast, 45.8% of medical students in a developing country used FTs to track physical activity^47^. In another study the prevalence of mobile health apps was 47.9%^45^. Additionally, studies have shown that 38.2% of respondents currently use FTs, while 19.3% have used them previously^21^and 57.5% of respondents in eastern Saudi Arabia owned a fitness watch or app^22^. FT usage has been linked to increased physical activity and reduced sedentary time^48,49^. The tracking features and goal-setting capabilities of FTs may contribute to this positive association. In addition, research suggests a positive correlation between FT use and higher exercise levels, supporting the benefits of FTs in promoting healthy habits^49^. However, the usage of FTs in our findings may be attributed to Saudi Arabia’s unique cultural and psychological landscape. Specifically, data privacy concerns and limited understanding of wearable technology’s benefits and applications might hinder adoption^12^. Nevertheless, Vision 2030’s initiatives, such as investing in digital infrastructure through 5G networks and promoting a digital economy, are likely to further support wearable technology adoption^12^.

### Types of fitness trackers and frequency of use

This study found that wristbands (43.7%) and smartwatches (35.9%) were the most commonly used FTs among university students. These findings differ from previous studies, which reported varying tracker usage patterns^3,20^. For instance, a US study found that 60% of students preferred Fitbit wristbands, followed by smartwatches (26.3%)^20^. Similarly, in Lebanon and Australia reported diverse brand preferences, including Fitbit, Apple Watch, and Garmin^3,25^. Specifically, 67.5% of Australian participants used Fitbit wristbands, while 16.5% used Garmin devices^25^. Regarding FT usage duration and frequency, our study revealed that 20.7% of students used FTs for 5–7 months, 17.4% used them for over a year, and 23% wore their trackers daily. Additionally, 21.6% wore their trackers most days of the week. These findings align with a US study, where participants wore their trackers “every day without fail” or “most days,” with many wearing them continuously, including during sleep^20^. A study in Australia found that respondents used FTs for 5–7 months and generally intended to continue usage^25^.

### Reasons for using fitness trackers and mobile health apps

This study found that healthcare students primarily used FTs and mobile health apps for tracking movements (39.2%), monitoring progress towards daily goals (16%), tracking active minutes (12.9%), calorie monitoring (14.6%), and goal-oriented reminders (11.8%). These findings align with international studies, which report similar FT usage patterns^3,20,25,45^. For instance, research among Lebanese university students found FTs were used for goal-setting, physical activity tracking, sleep monitoring, and calorie tracking^3^. A study among college students found that FTs were used for various purposes, including increasing physical activity (71.3%), step tracking (88.8%), distance tracking (87.5%), workout improvement, weight loss, sleep monitoring, and friendly competition^20^ Similarly, research in Thailand showed that individuals used FTs to track progress and stay informed^50^. In Australia, a study among adults reported that FTs were commonly used for step tracking (95%), active minute monitoring (76%), sleep tracking (66%), heart rate monitoring (63%), and tracking stairs climbed (58%) and energy expenditure (57%)^25^. In another recent study the main motives for using mobile health apps were to promote health status (68.6%) and to lose weight (33.2%)^45^. These studies collectively demonstrate that FTs facilitate self-monitoring of activity levels among students and individuals^50–52^. Our study found that 70% of students reported that FTs motivated them to engage in physical activity, and 20.7% reported an increase in daily step count. Moreover, 25.8% of students felt extremely confident and 28.6% felt moderately confident about being physically active after starting FT use. These findings align with a US study, where 61.3% of students reported being moderately confident and 23.8% extremely confident in increasing physical activity^20^. Furthermore, 58% of our study participants agreed that FTs boosted their motivation for physical activity, and 55.1% reported improved overall health. These results are consistent with previous research, where 68.8% of American students felt motivated to be physically active while wearing FTs^20^. However, another review suggested that while wearable technology users may experience short-term health benefits, motivation can wane over time, leading to discontinued use^53^. This highlights the need for further research on the long-term impact of FTs on health outcomes.

### Association between FTs usage student’s characters

In this study year of study and student’s athletic status is a significant factor influencing the adoption of wearable technology. Previous literature revealed that individuals with younger age, being a Saudi national and highly educated, and working participants, as well as those with chronic diseases were significantly associated with the use of FTs and mobile health apps^45^.In another study findings shows that smart watch wearing is significantly associated with, the availability of fitness apps^54^. In addition, factors like image, a personal value, were particularly important among students to wear the technology^54^. Although Wrist-worn trackers offer a convenient and potentially more accurate way to track daily steps compared to smartphone tracking, which may be less reliable due to inconsistent carry habits^55^. Wearable devices also provide additional benefits, such as monitoring heart rate, breathing rate, and walking pace^55^. Research suggests that FTs users are often already regular exercisers, utilizing these devices to enhance their routine or prepare for specific events rather than to initiate lifestyle changes [22]. However, for individuals transitioning to a more sedentary lifestyle or seeking to increase activity, FTs could serve as a valuable tool^55^.The main strengths of this study include its timely exploration of digital health trends among healthcare students, aligning with Saudi Arabia’s Vision 2030 initiative to leverage digital technologies and enhance healthcare delivery. The cross-sectional design provides a valuable snapshot of current perceptions and usage patterns, offering insights for educators, policymakers, and digital health stakeholders. Furthermore, by understanding healthcare students’ perspectives, the study identifies potential barriers and facilitators to digital health adoption, informing strategies to support the integration of digital technologies in healthcare.

### Limitations and recommendations

Although this study has limitations that should be taken into consideration. Firstly, only pharmacy, nursing, and EMS students from a single university in a specific region of Saudi Arabia were part of the sample, this narrows down the generalizability of the results to other healthcare students across the country, particularly those in urban or rural areas. One of the limitation is that homogeneity of the sample, since only selected specialization of the healthcare students were enrolled in the research. Therefore, to enhance representativeness, future research should aim for a more diverse and extensive sample, encompassing students from various disciplines and geographical locations in Saudi Arabia are needed. Secondly, self-reported information could potentially introduce response biases when completing surveys. Therefore, future studies should aim to enhance data diversity and gain a deeper understanding of the factors influencing the use of FTs by utilizing observational methods or conducting interviews (with appropriate consent). Such research is crucial in order to develop more targeted and effective interventions, and to comprehensively understand the impact of wearable electronic devices on healthcare outcomes. Although current findings have several implications for future interventions and their application, one suggestion is to develop user-friendly and affordable wearable technologies. Designing user-friendly interfaces and providing clear instructions can enhance adoption. Developing targeted awareness campaigns can promote effective use of wearable technology. Furthermore, providing Arabic language support can increase accessibility. Integrating wearable technology into healthcare systems can enhance patient care. Finally, targeted interventions are needed to promote adoption of wearable technology among students, healthcare systems, and other populations.

## Conclusion

Our findings underscore the potential of technology, particularly FTs and mobile health apps, in promoting health and wellness. Notably, FT use was associated with increased physical activity and enhanced confidence levels, highlighting the need for greater awareness among students and individuals about the benefits of FTs and mobile health apps. Moreover, our results indicate that year of study and athletic status significantly influence wearable technology adoption, with athletes utilizing FTs more frequently than non-athletes. Future research should investigate the motivations behind technology use for health improvement. Our findings can inform interventions aimed at creating awareness and beneficial effects of FTs and Mobile apps to boost health status. A longitudinal study tracking changes in usage patterns over time would provide valuable insights into digital health initiatives’ impact. Moreover, our results can help FT suppliers and manufacturers address usage barriers and improve product availability. Identifying vulnerable student populations and providing targeted education on FT benefits could be a valuable direction for future research.

## Data Availability

The data generated and analyzed in the current study are available from the corresponding author upon request.

## Abbreviations

FTs: Fitness trackers
WHO: World health organization
USD: United States dollar
CAGR: Compound annual growth rate
EMS: Emergency medical services
SPSS: Statistical package for social science
CI: Confidence interval
Apps: Applications
TAM: Technology Acceptance Model
HBM: Health Belief Model
